# The struggle towards ‘the New Normal’: a qualitative insight into psychosexual adjustment to prostate cancer

**DOI:** 10.1186/1471-2490-14-56

**Published:** 2014-07-30

**Authors:** Narelle Hanly, Shab Mireskandari, Ilona Juraskova

**Affiliations:** 1 Faculty of Health Sciences, The University of Sydney, Sydney, NSW, Australia; 2 Centre for Medical Psychology & Evidence-based Decision-making (CeMPED), School of Psychology, The University of Sydney, Level 6, Chris O’Brien Lifehouse (C39Z), Sydney, NSW 2006, Australia

**Keywords:** Prostate cancer, Sexual function, Self-perception, Qualitative research

## Abstract

**Background:**

Despite the growing body of literature which highlights the potential for significant and enduring side-effects of prostate cancer treatment, there is limited research exploring the experience of living with the treatment-induced side-effects such as sexual dysfunction, and their repercussions for men and their partners. The aim of this qualitative study was to explore factors influencing psychosexual adjustment, self-perception, and unmet information and support needs of prostate cancer patients and their partners.

**Methods:**

Twenty-one men, recruited via a prostate cancer support group newsletter, participated in face-to-face semi-structured interviews, which were subjected to thematic analysis.

**Results:**

The qualitative analysis revealed three inter-connected main themes which contributed to men’s psychosexual adjustment: i) Psychosexual impact, ii) Communication and support, and iii) Integration process. Men reported distressing sexual and urinary difficulties, tainted self-perception and altered intimate relationships. Receiving adequate information and support, and having good communication with their doctors and partners facilitated better adjustment to prostate cancer treatment. Coming to terms with the significant impact of treatment had involved making lifestyle changes, coping with emotional struggles and striving to accept and integrate their post-treatment “new normal” self and sexual life.

**Conclusions:**

The importance of adequate communication with health professionals and partners, especially regarding treatment effects on sexual function and rehabilitation options, was highlighted as a key factor facilitating the adjustment process. Prostate cancer patients would benefit from improved access to timely and tailored information and decision-making resources, ongoing multidisciplinary care, and support groups, as well as appropriate referrals for sexual and psychological counselling.

## Background

In 2009, 19,438 cases of prostate cancer (PC) were diagnosed in Australia [1], with a five-year survival rate of 92% [2]. Following diagnosis, men are presented with a number of treatment options, including: i) observation without invasive treatment, with a view to intervene in the event of disease progression (i.e. active surveillance or watchful waiting); ii) removal of the prostate (RP – radical prostatectomy, nerve sparing or non-nerve sparing, delivered via open, laparoscopic, or robot-assisted surgery); or, iii) radiotherapy (RT – external radiation or brachytherapy). In addition, hormone therapy (ADT – androgen deprivation therapy) may also be offered alongside another mode of treatment for prostate cancer as adjuvant therapy [3]. Survival rates are similar across treatment options [4].

At 3 years post-treatment, urinary dysfunction (e.g. urinary incontinence) is more common amongst men treated with radical prostatectomy (up to 15%), and bowel problems (e.g. faecal incontinence and bleeding from the bowel) are more commonly reported by men treated with radiotherapy (up to 15%). Sexual dysfunction three years post-treatment is common across all modalities (nerve sparing RP 68%, non-nerve sparing RP 87%, external RT 68%, brachytherapy 36%, ADT 98%) [4]. Treatment-induced changes in sexual function include erectile dysfunction (ED), absent or diminished ejaculate, changed orgasmic sensation, urine loss during arousal or orgasm (climacturia), decreased libido and penile shortening [5-7]. In addition to these physical side-effects, men also report impaired body image and self-esteem [4,8,9]. ADT may also result in fatigue, weight gain, loss of muscle mass and body hair, hot flushes, sexual dysfunction, diminished genitalia size, depression, mood swings and reduced cognitive function with associated decline in quality of life [10]. Given the high survival rate, the majority of men diagnosed with PC are living with these physical and psychological consequences of treatment which have persistent effect on their quality of life [4].

Until recent years, how men felt about the impact of treatment side-effects was not addressed in any detail [11]. Sexual function was usually evaluated quantitatively, in terms of erections and the ability to achieve vaginal penetration, with much less emphasis on diminished desire and intimacy [12], and the impact of sexual problems on relationships [13]. Current research which recognises the broader psychosexual impact of treatment-related functional changes has found that many men report a negative association between physical side-effects and their intimate relationships [14,15], and self-perception [16], including reduced quality of sexual intimacy, decreased sexual desire and sexual confidence, impaired feelings of masculinity, lower self-esteem and poorer body image [16,17].

It is not surprising then that post-treatment side-effects have been found to increase anxiety and depression [4,8,9,18] which can impact on already reduced sexual function [19,20]. Men may also grieve for their diminished sexual quality of life, decreased libido and lost sexual fantasy life, as a result of their decreased self-worth and a depleted view of their masculinity [21].

Studies have shown that after PC treatment, men express unmet needs related to sexuality. Smith et al. [22] found that 47% of participants in their study described unmet needs related to changes in sexual feelings and the associated impact on relationships. In contrast, men who believe they received adequate information to enable informed treatment decision-making have been found to be less likely to report being unhappy with their doctor or distressed about side-effects [23,24].

Further investigations of men’s experiences of treatment-induced side-effects is required to gain a better understanding of the issues underlying the complex post-treatment changes in the men’s lives. Such deeper understanding can then inform the development of much needed tailored interventions to assist men in coping with post-treatment changes in sexual function and activity, as well as self-identity. The aim of the current study was to further explore the experiences of men treated for PC and their psychosexual adjustment, as well as identifying unmet information and support needs.

## Methods

This study was approved by the ethics committees at the University of Sydney and Concord Repatriation General Hospital. An advertisement was placed in the Concord Hospital Prostate Cancer Support Group newsletter inviting men who had been diagnosed and treated for PC in the past 5 years to participate. Men younger than 18 years, with insufficient English to understand and give informed consent and participate in the interview, or who had concurrent malignancy (of another type) and/or psychiatric disorder, were excluded from participating in the study. Phone screening for eligibility was conducted for self-selected men who responded to the study advertisement.

All eligible men participated in an individual, face-to-face, semi-structured interview with the first author (NH). On average, the interviews lasted approximately 1.5 hour (range = 50 minutes to 2 hours). An *aide-memoire* was used to structure the interview, outlining the major questions and topics to be covered during the interview, whilst leaving the wording and sequencing of questions open. Questions were designed with detailed probes targeting patient experiences in a number of areas which covered: pre-treatment knowledge of potential treatment-related side effects, including discussion of side effects with clinicians; experience of sex and sexual activity pre-treatment and at the time of interview, changes in sexual life since treatment, management of changes in sexual life post treatment, effects of treatment on quality of life, impact on existing relationship or on ability of single men to seek a relationship, coping strategies and information provision prior to treatment. These broad areas were chosen on the basis of a review of the literature and the consensus among the authors based on their extensive expertise. All participants provided written informed consent with guarantees of confidentiality.

The interviews were audio-taped, transcribed and subjected to thematic analysis in accordance with Braun and Clark’s [25] methodology: i) familiarisation with the data, ii) code generation, iii) searching for themes, iv) reviewing themes, v) defining and naming themes, and vi) producing the report. Through the first stage of open coding, the data was grouped into smaller segments with a descriptor or ‘code’ attached to each segment; followed by axial coding, which entailed the codes being grouped into similar categories. These features were checked for emerging patterns, variability and consistency and commonality across participants until saturation (i.e. when analysis produced no new themes or categories) was reached. Throughout the iterative analysis, researchers (NH and IJ) discussed the key features of the data, enhancing researcher sensitivity and overcoming selective inattention.

## Results

### Demographic and medical characteristics

Of the 30 men who responded to the advertising, 3 men did not meet the eligibility criteria as they were more than 5 years post-treatment. A further 6 men opted to withdraw from the study, citing: partner’s reluctance for them to participate (n = 2), believing they could not provide any useful information (n = 1), and no reason given (n = 3), resulting in 21 completed interviews (response rate 78%).

Demographic and medical characteristics of participants are summarized in Table 1. About two-thirds of the participants were aged 60–69 years at the time of the interview (62%), and more than half were aged 50–59 years at the time of treatment (52%). The majority of participants were married (76%), heterosexual (95%), had post-school qualifications (62%) and approximately half worked full-time (52%).

**Table 1:** Demographic and medical characteristics of participants (N = 21)

**Current age (years)**	**n**	**%**
50-59	8	38.1%
60-69	13	61.9%
**Age at Treatment (years)**		
<49	1	4.8%
50-59	11	52.4%
60-69	9	42.9%
**Marital Status**		
Married	16	76.2%
Single	2	9.5%
Divorced	2	9.5%
Relationship (not living together)	1	4.8%
**Sexual Orientation**		
Heterosexual	20	95.2%
Homosexual	1	4.8%
**Education**		
Year 10 or less	7	33.3%
Year 12	1	4.8%
>Year 12	13	61.9%
**Employment**		
Full-time	11	52.4%
Part-time	2	9.5%
Retired	7	33.3%
Unable to work	1	4.8%
**Treatment**		
Radical Prostatectomy	19	90.5%
High Dose Brachytherapy and ADT	2	9.5%
Radical prostatectomy		
Plus radiotherapy	1	4.7%
Plus radiotherapy and ADT	1	4.7%
Plus ADT	2	9.5%
**Time Since Treatment**		
≤ 5 months	2	9.5%
5-12 months	4	19%
≤2 years	7	33.3%
≤3 years	6	28.6%
≤5 years	2	9.5%

ADT – Androgen Deprivation Therapy.

All 21 participants had been initially diagnosed with localised prostate cancer amenable to treatment with curative intent. Nineteen participants (90%) underwent radical prostatectomy with a further 2 participants (10%) treated with high dose rate brachytherapy (HDRB) and ADT (one had completed ADT and one nearing completion). At the time of their interview, 4 (19%) participants had undergone further treatment for localised disease progression, with 1 undergoing RT, 1 RT and ADT; and 2 treated with ADT alone. Six (29%) men were within the first 12 months since initial treatment, 13 (62%) were less than 3 years, and 2 (9%) were less than 5 years since treatment.

### Thematic analysis

**Figure 1:**
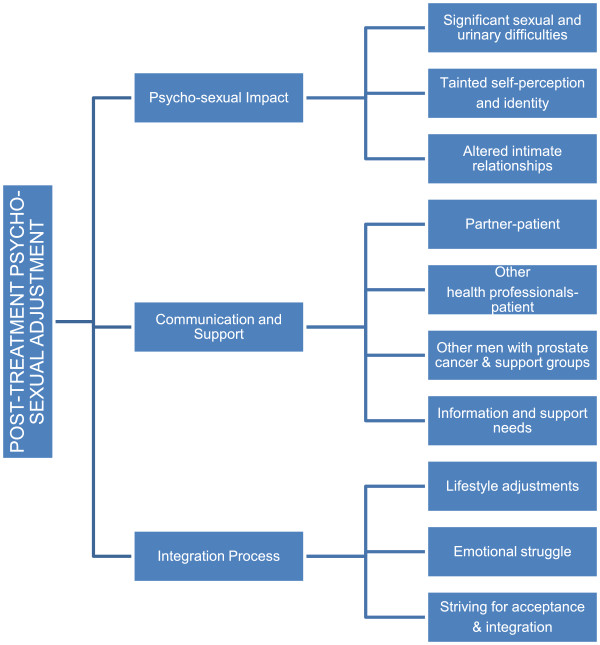
Qualitative analyses themes and sub-themes for post-treatment psychosexual adjustment to prostate cancer.

Analysis of the participants’ interviews revealed three main themes relating to men’s post-treatment psychosexual adjustment: i) psychosexual impact, ii) communication and support, iii) integration process; with a number of sub-themes emerging in each main theme as illustrated in Figure 1.

#### Theme 1: psychosexual impact

##### Significant sexual and urinary difficulties

Physical changes reported by participants included erectile dysfunction, urinary incontinence, urine leakage during arousal or at orgasm, reduced penile size, lack of or reduced ejaculate, change in intensity of orgasm, reduced desire and pain.

The inability to achieve and/or maintain an erection and therefore have penetrative intercourse precluded some participants from sexual intimacy, as many viewed sexual interaction as equating with penetrative intercourse.

“…minimal erections, so there’s no penetration....The major issue has been around penetration and the inability to be able to get an erection.” [age 65]

Some men noticed that, unlike previously when erections had been spontaneous, they now consciously thought about what was happening during arousal, thinking about how long their erection would last, what their partner was thinking and whether they could “perform” – all of which further negatively impacted arousal and sexual experience.

“You do think about it [erection]… and that probably takes away from the moment.” [age 59]

“…when it does come to having, coming close to – whether it be penetration or just physical foreplay, I get very tense or just lose interest completely.” [age 51]

Changes in orgasm including diminished intensity or even complete absence of orgasm, lack of or reduced ejaculate, negative changes in emotional experience of orgasm, and changes in libido and desire were also reported.

“I don’t have sexual difficulties because I don’t have any sexual urges. When I say that, my wife and I aren’t intimate, we cuddle but that’s about it, cuddle and kiss.” [age 61]

“You feel a bit detached… It’s a bit like doing it with someone else’s dick… the feeling dies off quickly and there’s no “afterglow” like there was before.” [age 59]

Many men reported that the side effects of ED therapies, their cost and the effort required to use them were prohibitive. Additionally, ED therapies did not work for some men or were not an acceptable option (e.g. penile injections).

“With Viagra I can actually partly engorge my penis in a crouching position … I bought a vacuum kit with the rubber bands so I can achieve enough of an erection to have penetrative sex …it’s a bit of a production.” [age 62]

Urinary incontinence and ongoing urine leakage was a significant issue for a number of men. One participant refused to use pads and wore dark clothes to disguise wet patches, yet described leakage-related embarrassment. A participant explained how it affected his quality of life:

“[Urinary incontinence] stopped me…before I used to do a lot of walking. But one of the things it really inhibited was going for a swim. Because you can’t swim with a pad on and you can’t go to the beach with your pants starting to get wet all the time.” [age 62]

Urine leakage during arousal and/or at orgasm was found to be a difficult and embarrassing issue, sometimes leading to avoidance of physical intimacy for these men and in some instances, for their wife/partner.

“…a couple of times it [urine leak] happened and [my wife] got so angry, she jumped up and went and had a shower and I felt terrible…she refused to even talk about sex anymore…can’t blame my wife and I couldn’t blame myself because you just can’t control it.” [age 58]

##### Tainted self-perception and identity

Several men reported PC and its related physical changes had significantly affected their self-perception and self-esteem. Sexual difficulties resulted in feeling less confident, particularly for single men some of whom chose not to engage in new social interactions with potential for intimacy. Men described feelings of inadequacy and embarrassment due to urinary leakage, ED and smaller genitalia.

”I think my impression of myself now as a man is a bit lower than before I had that operation.” [age 60]

"It’s very hard to put it into words… it [being able to have erections] is part of your self-image, your self-confidence…when it [ED] happens it really does change you.” [age 54]

You’re suddenly different, non-performing… you’re almost like …the eunuchs …you feel like you’ve been neutered almost. A normal healthy, heterosexual male as far as I know, feels that [erection] is a powerful thing for him and to have it taken away, takes a bit of you away.” [age 54]

Impotence was found to have a deep impact on self-perception, above and beyond sexual interactions. Lowered self-esteem due to impotence was reported by one man as affecting his confidence to four-wheel drive in the same pre-treatment “aggressive” manner.

“The self-esteem gnaws away at you in really unusual situations…sexuality is important but self-esteem and self-confidence tends to hang off that issue.” [age 62]

Men who reported a good response to medical therapy to achieve erections reported the additional benefit of restored confidence in themselves as males.

“Since having the needles it’s given me a lot more confidence…It’s a lovely feeling, you feel very proud of yourself. You put the needle in and everything’s working again. Being a selfish male and my ego....” [age 58]

##### Altered intimate relationships

In general, men described their partners as being supportive in terms of diagnosis and treatment, even if issues related to treatment side-effects were problematic. Most married men reported that their diagnosis and treatment had an impact on their relationship with their wife. In some cases, men reported a positive impact of PC diagnosis on their relationships:

“I do feel that I’m in a very loving relationship and I am very well loved. I think that has always been the case but it is probably more so after the operation.” [age 62]

”It is not just the sexual stuff, it’s about your feelings for each other. As a man, it’s not just about satisfying yourself but it’s about making sure that you’re satisfying each other.... that’s with the sexual aspect, the emotional aspect, the communication. I find myself cuddling my wife a lot more often. I find myself being very considerate and watching her and anticipating…I’m more in tune with maybe how she’s feeling or how she’s responding now.” [age 53]

Some men worried about their partners and how they were affected by the changes in their sexual functioning.

“… I thought that the fact that I couldn’t make love properly anymore had affected my wife. She says it didn’t but I know. She even asked her sister how many times they make love… and I’m thinking, this was an issue for her.” [age 58]

For men who defined sexual intimacy as penetrative intercourse, lack of erections meant no sexual intimacy.

“It doesn’t worry me not having sex - we’ve discussed it that there’s nothing we can do, so there’s no sex life in our place…there’s no sexual intimacy.” [age 61]

Some partners who were otherwise supportive were not always willing to try non-penetrative sexual activity, particularly if such activities had not previously been part of their sexual repertoire.

“I’m not comfortable with things like that [outer-course, manual stimulation or oral sex]…they are not an option for her and I.” [age 61]

A gay participant’s embarrassment due to ED and urine leakage led to reduced socializing and significant reduction in sexual encounters.

“You take headache tablets before the Caverject and have a wee…and have a towel in bed. It’s always been spontaneous…pick someone up and go home with them…I can’t go to their place, I’ve got to think of what I’ve got to do so they can’t see what I’m doing.” [age 67]

Understandably, couples who had pre-existing relationship and sexual issues appeared to face greater challenges adjusting to treatment side-effects.

#### Theme 2: communication and support

Men who reported they had good communication with their partner, doctor and other health professionals reported better adjustment to PC, especially regarding sexual outcomes.

##### Doctor-patient

Men reported great trust in doctors who provided them with adequate information and ample opportunity to ask questions.

“I wrote all the questions and asked him [doctor] when I went in there and he said ‘don’t worry about the other people in the waiting area you’re in here now and I will take the time to answer all your questions’ and he did.” [age 69]

Some men noted that they felt uncomfortable talking about sexual issues and if their doctor did not raise the subject, neither did they.

“He didn’t tell me the [sexual] after effects and to be quite honest I didn’t ask.” [age 61]

A number of participants noted that their doctor raised the topic of sexual function often which was appreciated, particularly if the man was uncomfortable having that particular conversation.

“Honestly, my urologist said ‘why I keep bringing it up is because some people won’t talk about it [sexual side-effects]’ and I suppose that’s right.” [age 63]

A few men reported their urologist had failed to pick up on the emotional cues indicating emotional distress, and hence they sought help themselves elsewhere.

“With the resultant depression I became extremely distressed and morose and … I asked my GP to refer me to a psychologist which he did.” [age 69]

##### Partner-patient

Communication difficulties which existed for some couples prior to diagnosis were compounded by problems associated with PC. Couples who had previously communicated well on difficult issues reported communicating similarly well about difficulties associated with treatment.

“Well, what you get afterwards is only a product of what’s been going on beforehand isn’t it?” [age 54]

Some men discussed hiding their need to use some ED treatments from their partners.

“I think my wife would be absolutely shocked if she knew. I think that deep down when she found out I was taking these tablets [Viagra], she wasn’t happy with that, and I think if she found out I was having injections – geez. I haven’t told her about the injections, I’ve sort of kept that one to myself.” [age 60]

Some participants explained that while medical procedures were discussed with their partners, the possible impact on their sexual life had not been discussed.

“Even though I talked about the treatment, what I was going through, and wanting to find the doctor that could ensure erections after the operation, we didn’t actually talk about what will happen after the operation. She [wife] knew what I was going through, she knew why I was going to see all these different doctors and all this, but we didn’t actually sit down and say ‘ok, when the operation takes place and whatever happens afterwards, what’s it going to be like?’ We didn’t talk about that.” [age 59]

##### Other health professionals-patient

Out of 21 participants, nine had been referred to another health professional (such as specialised prostate cancer nurse, psychologist) for support at the time of diagnosis and all the men reported this as being very helpful.

“Talking to the nurse at the hospital and the staff from the Cancer Council Telephone Support Group, talking to the psychologist …I got the message that ’there is light at the end of the tunnel, there is hope’.” [age 53]

##### Other men with prostate cancer and support groups

Men who had attended a support group described it as a reliable source of information and a confidential and safe opportunity to discuss their concerns.

“Those meetings are pretty good. I understood more about things…I learned a lot really. And it’s good to hear other people’s problems and what they had”. [age 60]

##### Information and support needs

Those who had access to adequate information, felt they were better prepared for treatment side-effects and found it easier to adapt to ongoing changes. Some participants felt they should have received more information about side-effects, the likelihood of their occurrence and available management strategies. A frequent comment was that in the early phase of their diagnosis men did not know enough about prostate cancer and its effects to know what questions to ask.

“I sat in the hospital and didn’t know what was going to happen to me. It’s alright to say ‘oh, you’re going to have an operation’ but what does it mean.” [age 61]

For some men potential sexual rehabilitation options were not discussed in any detail by their doctor and they felt uncomfortable initiating the discussion.

“It was just in passing one day [the doctor] said to me, ‘Oh, why don’t you try this drug. It’s better than Viagra’ and of course it had no effect. And then about a year later he said ‘oh, why don’t you try the Caverject’.” [age 58]

Most men believed early referral for ongoing support from other health professionals other than their doctor (e.g. nurse, psychologist), would have reduced their anxiety and improved their understanding of treatment procedures, side-effects and management options.

“I think there probably does need to be an opportunity, away from the surgeon, for discussion about those physical details in more detail than I received.” [age 62]

Some men commented that health professionals should provide men and their partners with information regarding feelings they may experience, and encourage and support couples to explore sexual activities other than penetrative intercourse.

#### Theme 3: integration process

##### Lifestyle adjustments

To accommodate the functional changes resulting from treatment, many men reported making positive lifestyle changes such as improved diet and exercise which had lead to better overall general health.

“One of the big benefits that’s come out of it is that I now really do look after myself.” [age 63]

Men with post-treatment urinary incontinence discussed lifestyle changes such as reducing general fluid and alcohol intake, and planning activities and travel around availability and access to toilets.

##### Emotional struggles

Living with the consequences of PC and its treatment was difficult for many of the participants who described experiencing emotions such as shock, anger, depression, disappointment and a sense of loss associated with ongoing changes in sexual function, penile shortening and loss of libido.

“And it’s hard sometimes, some days it’s very hard. And you get disappointed but you know, you’ve got no choice.” [age 58]

“I don’t have crying fits, I used to, but I don’t now.” [age 61]

In some instances struggling with PC had acted as a catalyst for psychological distress around issues unrelated to cancer diagnosis e.g. retirement. Men with progressive disease reported greater emotional impact and more difficulties adjusting to ED and incontinence.

##### Striving for acceptance and integration

Not all the men felt they had successfully accepted the changes following treatment and reported believing that it is an ongoing process.

Men described using various coping strategies from the time of diagnosis to living with post-treatment side effects. Denial behaviour was discussed by some men such as not reading information provided, or leaving the room while doctors were providing information. Some men reported minimising the occurrence/impact of possible sexual side-effects, as they believed they would not suffer any side-effects, or they would not be significant and they would be able to adjust.

“I really didn’t think about it [ED] then. I thought I was very strong and fit, would get through it and it wouldn’t really affect me. I was very positive that ‘well, I’ll get over it and there won’t be any problems with me and if they’re minor, I’ll adjust to them’.” [age 58]

A few men engaged in unhelpful behaviours such as excessive alcohol intake despite the potential for embarrassing urinary leakage.

In contrast, a number of men discussed that their general positive attitude had helped them through the difficulties they had experienced.

“I guess it’s a mindset that says ‘today is going to be a new day’. I’m better than what I was before.... I have a positive aspiration.” [age 53]

Some reported that talking to other men who have been through treatment and to experienced PC health professionals, helped in accepting the post-treatment changes and ‘getting on’ with their life after cancer.

Some men reported they had re-evaluated aspects of their life and this had helped them adjust to their new circumstances.

“When things finally settle down and you think ‘this is what it’s going to be like forever’ then you accept it. I came across that [concept] on one of those health shows on TV, talking about people having to accept ‘the new normal’ and I thought that’s a lovely phrase. I could relate it to me.” [age 58]

## Discussion

The aim of the current research was to explore men’s experiences of PC and the impact of treatment-induced changes on men’s sexual life, self-perception, as well as their intimate relationships. Three themes underlying the men’s post-treatment adjustment were found: psychosexual impact, communication and support, and integration process, each of which was comprised of a number of sub-themes. Participants discussed significant physical, functional, psychological, emotional and relationship changes as a result of PC treatment which affected their sexual life, self-perception and relationship. The importance of receiving adequate information from treating doctors and having good communication with their doctors, partners and other health professionals were highlighted as major contributors to better adjustment for men in this study.

The significant sexual functioning issues reported by the participants reflect previous research findings (e.g. Dahn et al. [17]) emphasising the need for patients to be well-informed about treatments for erectile dysfunction and to be offered sexual counselling. Men in this study appeared to benefit from discussions and interventions for treatment side-effects, particularly those affecting sexual and urinary functioning. However, if both men and their treating doctors are uncomfortable discussing sexual function in any detail, men may be deprived of the opportunity to better understand and better manage their erectile dysfunction [26]. Hence, men with PC (and their partners) would benefit from access to multidisciplinary sources of care, including prostate nurse-led psychoeducational sessions and psychological care, as well as access to support groups. An important point was made by Wootten et al. [27] who found men’s management techniques are more emotion-based in relation to sexual dysfunction and more problem-focused with regard to incontinence and suggest this may be a consequence of inadequate information being provided about the practical management of sexual dysfunction.

Participants discussed the impact of post-treatment changes in sexual function on their partners and intimate relationships. The level of concern of patients on their sexual function may not be shared to an equal degree by their partners [28]. Indeed, PC patients and their partners have been found to differ on sexual-related measures [29]. While patients express unease with the sexual side-effects of their cancer treatment and this is a significant area of concern, their female partners recognize decreased sexual desire in their partners post-treatment, but this is not considered a primary concern [30]. As greater sexual dissatisfaction has been found to be associated with poorer marital adjustment in couples who reported low levels of communication, psychosocial interventions that facilitate healthy spousal communication and address sexual rehabilitation needs of both the patients and their partners after PC treatment seem essential [31]. Hence, the partners’ involvement in sexual rehabilitation is warranted.

In addition to addressing sexual functioning, understanding the impact of PC treatment on the masculine gender role may facilitate better understanding of men’s adjustment following treatment [32]. Male sexual potency is seen as an important social masculine trait [32]. Hence, it is not surprising that for many men with PC, the loss of sexual function is associated with feelings of tremendous despair, as it may disrupt the identity of men who define masculinity through sexual performance [32]. Interventions may focus on promoting a more flexible gender schema such as a view of men’s identity as not being limited to their sexual performance, to allow better adaption to diminished sexual function [33].

Steginga et al. [11] found that 25% of 206 men in their study who had been treated for PC continued to report moderate to high unmet needs for information related to investigations, treatment options, side-effects and their management. Receiving written information pre-operatively, no matter how comprehensive, is not sufficient to foster the patient’s management of all post-operative consequences of PC [34]. Personal follow-up support such as telephone follow-up calls has been found to facilitate better adjustment after surgery [34]. Christie et al. [35] found that discussions about treatment options with people from the patients’ social networks, prior to beginning treatment, significantly contributed to improvements in affect for patients at1 and 6 months following treatment, while discussions with physicians predicted an increase in positive affect 1 month following treatment.

The limited research on psychosocial interventions for men with PC points to significant improvements in the men’s psychosexual adjustment [36]. Group cognitive-behavioural and psycho-education interventions have been found to be helpful in promoting better psychological adjustment and quality of life (QOL) for patients; while coping skills training for couples has been found to improve QOL for partners [36]. A counseling intervention aimed at improving levels of sexual satisfaction and increasing successful utilization of medical treatment for ED for patients and their partners was found to significantly increase sexual function and satisfaction [37]. A computer-assisted, nurse-led intervention focusing on providing education and support resulted in long-term improvements in quality of life outcomes related to sexual functioning and cancer worry [38]. A randomized controlled trial of a 10-week group-based cognitive–behavioral stress management intervention with older men who had undergone a radical prostatectomy was found to be highly effective in promoting sexual recovery [39]. It is anticipated that the obtained findings will inform future research to identify the best practice model for the development of a psycho-educational intervention for men/couples from diagnosis through treatment, recovery and rehabilitation. It is hoped that provision of such an intervention early in the disease trajectory will lead to improved quality of care and quality of life of men affected by PC and their partners/families.

### Limitations

The study population was self-selected from a newsletter mailing list of a Prostate Cancer Support Group, coordinated by the researcher (NH). Therefore self-selection bias may characterise this sample. Since the sample consisted of men for localised or locally advanced PC and 19 of 21 participants underwent radical prostatectomy, the findings may not reflect experiences of men with metastatic PC and those undergoing other treatment types. Further, the interviewer’s female gender may have potentially affected participants’ responses given the intimate nature of the subject matter. However, participants’ appeared candid when discussing the impact of side-effects on their QOL and their relationships. A final interview question asked whether anything would have made the men feel more comfortable during the interview and all responded they were comfortable. In addition, given that the interviewer was also the participants’ support group coordinator, the impact of this on the participants’ answers cannot be known. Some participants explained that they preferred to be interviewed by the coordinator whom they knew rather than a stranger.

## Conclusions

A timely provision of information and ongoing multidisciplinary support to men diagnosed with prostate cancer and their partners will afford them a better understanding of the potential impact of side-effects, particularly related to sexual function, on their personal and social relationships, their body image, self-esteem and sexuality. By identifying potential issues early and providing relevant information and support, men will be better prepared to accept and adjust to the post-treatment changes in their sexual function. Future research is recommended to examine a nurse-led information and psychosocial support role and to confirm the benefits reported by participants in this study and identify other potential benefits or otherwise. It should also identify appropriate content, optimum timing and duration of a nurse-led information and psychosocial intervention to develop the best practice model of care for tailoring interventions for men with PC, thereby providing long-term benefits to men and their partners adjusting to changed sexual function after treatment. A multidisciplinary approach to PC care whereby clinicians, nurses, allied health professionals as well as peers work together to provide appropriate information and support over time seems needed in order to adequately address the complex adjustment process of men with PC and their partners.

## Abbreviations

PC: Prostate cancer; RP: Radical prostatectomy; RT: Radiation therapy; ADT: Androgen deprivation therapy; ED: Erectile dysfunction; QOL: Quality of life.

## Competing interests

Narelle Hanly, Shab Mireskandari and Ilona Juraskova declare that they have no conflict of interest or competing interests.

## Authors’ contributions

NH conceived and coordinated the study, conducted the interviews, analysed the data and drafted the manuscript. SM helped to draft the results and the manuscript. IJ supervised the study, helped with the analysis and write up of the results and the manuscript. All authors have read and approved the final manuscript.

## Pre-publication history

The pre-publication history for this paper can be accessed here:

http://www.biomedcentral.com/1471-2490/14/56/prepub
